# Symptomatic Brain Radiation Necrosis in an Anaplastic Lymphoma Kinase (ALK)-Positive Non-small Cell Lung Cancer (NSCLC) Patient After Fractionated Stereotactic Radiotherapy While on Alectinib

**DOI:** 10.7759/cureus.35952

**Published:** 2023-03-09

**Authors:** Sitaraman BalajiSubramanian, Thuraya Al Hajri, Namrata Satyapal, Simin Laiq, Zahra Al Hajri

**Affiliations:** 1 Department of Radiation Oncology, Royal Hospital, Muscat, OMN; 2 Department of Pathology, Khoula Hospital, Muscat, OMN

**Keywords:** alk-positive, non small cell lung cancer, alectinib, radiation necrosis, fractionated stereotactic radiotherapy, alk-positive non-small cell lung cancer, brain metastases

## Abstract

Anaplastic lymphoma kinase (ALK)-positive non-small cell lung cancer (NSCLC) has a higher incidence of brain metastasis. Despite having a favorable prognosis and relatively long survival with second-generation ALK tyrosine kinase inhibitors (TKI), patients can have substantial morbidity, negatively affecting functional progression-free and symptom-free survival. Studies have shown that ALK-rearranged NSCLC is a risk factor for developing radiation necrosis (RN). Recently, second-generation TKI, especially lorlatinib, alectinib, and brigatinib, have demonstrated good central nervous system (CNS) penetration and overall response rates in patients with brain metastasis. However, to improve overall outcomes in symptomatic or limited brain metastases, stereotactic radiosurgery (SRS) is increasingly preferred over whole brain radiotherapy (WBRT) prior to systemic therapy to avoid significant cognitive deterioration. To improve the therapeutic ratio, fractionated stereotactic radiotherapy (FSRT) has been explored for brain metastasis. Herein, we report on one ALK-rearranged NSCLC patient who developed RN despite FSRT, one year after the completion of radiotherapy while on alectinib.

## Introduction

Approximately 5% of non-squamous non-small cell lung cancers (NSCLC) have anaplastic lymphoma kinase (ALK) gene rearrangements [1]. In patients with ALK-positive NSCLC, more than 50% may develop brain metastases during their disease course [2]. The ALK mutation is often referred to as the "Diamond Mutation" due to the good overall response and long-term survival of those treated with ALK tyrosine kinase inhibitors (TKI). For ALK-rearranged NSCLC, the one-year cumulative incidence of radiation necrosis (RN) is around 17.3% [3]. Second-generation TKI has good central nervous system (CNS) penetration and overall response rates in the brain and is increasingly being considered upfront in patients with asymptomatic brain metastases. However, studies have shown that stereotactic radiosurgery (SRS) prior to TKI improves overall treatment outcomes in symptomatic, limited brain metastases [4, 5]. SRS is preferred over WBRT due to the increased risk of cognitive decline when whole brain radiotherapy (WBRT) is combined with second-generation TKI [6]. Increasing refinements in radiotherapy machines, planning software, and image-guided devices enable stereotactic intracranial radiosurgery on systems such as linear accelerator-based SRS (LINAC-SRS), cyberknife, and gamma knife.

Clinical and dosimetric studies have compared LINAC-based systems with other radiosurgical systems in the upfront or salvage setting for intracranial and extracranial lesions [7-10]. However, comparing various SRS systems for various neurological indications remains an active research topic. Studies exploring fractionated stereotactic radiotherapy (FSRT) have reported improved therapeutic ratios even in smaller brain metastases by reducing the risk of brain RN while improving local control by increasing the biologically effective dose (BED) [11, 12]. While FSRT has shown encouraging results in preventing brain RN, we report a case of RN in a patient with ALK-rearranged NSCLC despite FSRT, one year after the completion of radiotherapy while on alectinib.

## Case presentation

A 41-year-old gentleman, a non-smoker from Oman, presented with an unrelenting cough of four months' duration and a weight loss of two months duration. On evaluation, he was diagnosed with a left lower lobe lung mass with malignant pleural effusion. Pleural fluid was hemorrhagic, and cytology was positive for adenocarcinoma. The patient was started on palliative chemotherapy (carboplatin, pemetrexed, and bevacizumab) in June 2021. In the last week of July, following the completion of two cycles of chemotherapy, he developed an intractable headache that was unresponsive to analgesics. A brain MRI showed a left frontal intra-axial heterogeneously enhancing mass lesion measuring 1.7 x 1.3 x 1.4 cm with significant surrounding edema.

Meanwhile, the molecular profiling of the lung mass was positive for ALK by fluorescence in situ hybridization (FISH) and negative for the epidermal growth factor receptor (EGFR), the ROS1 mutation, and programmed cell death protein 1 (PD-1) or programmed cell death ligand 1 (PD-L1). Considering the fact that the patient was symptomatic and ALK mutation-positive, he was offered FSRT instead of single-fraction SRS after a multidisciplinary tumor board (MDT) discussion comprising a neurosurgeon, neuroradiologist, medical oncologist, and radiation oncologist. In addition, the patient was started on steroids to reduce edema, which was tapered over two weeks.

**Figure 1:**
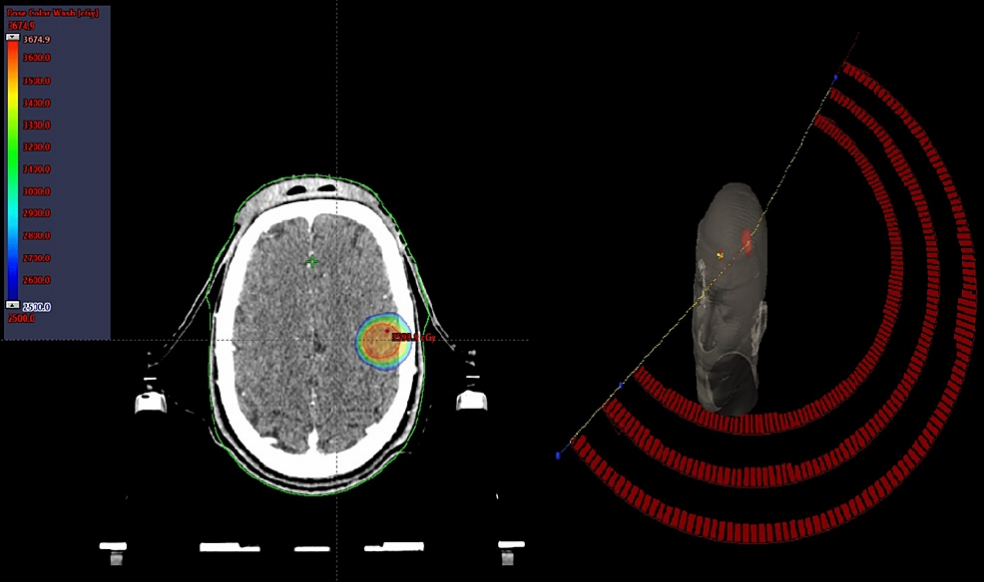
Dose distribution and beam arrangement of the fractionated stereotactic radiotherapy plan

The patient was immobilized in the supine position with a stereotactic thermoplastic mask before undergoing a thin-slice-enhanced MRI and contrast-enhanced CT scan. A slice thickness of 1.0 mm was used. The MRI and the simulation CT scan were performed to improve target delineation and identification of the organs at risk (OAR). The definition of gross tumor volume (GTV) was the enhancing abnormality confirmed on the MRI and CT scans with the T1 post-contrast sequence. The planning target volume (PTV) was generated by the geometric expansion of GTV plus 2 mm to compensate for uncertainties. The total prescription dose was 35 Gy in five fractions volumetrically, such that the entire prescription dose was applied to at least 99% of the PTV and delivered over a week in August 2021, three weeks after the second cycle of chemotherapy. The volumetric modulated arc therapy (VMAT)-based plan was optimized in the Eclipse treatment planning system (TPS) using 6 MV flattening filter-free beams for three coplanar partial arcs on a Truebeam STx (Varian Medical Systems, Palo Alto, California) equipped with a high-definition multileaf collimator (HDMLC) with 120 leaves. All doses were calculated using an anisotropic analytic algorithm with a grid size of 1.25 mm. In addition, as part of our brain metastasis radiosurgery program, kilo-voltage cone beam computed tomography (kV-CBCT) and Brain Lab ExacTrac X-ray imaging were used to ensure patient setup accuracy, and repositioning was provided in six dimensions using an integrated six degrees of freedom treatment couch. Figure 1 shows the dose distribution and beam arrangement of the fractionated stereotactic radiotherapy (FSRT) plan.

As a precautionary measure because second-generation TKIs are associated with an increased incidence of RN, a gap of two weeks was given following radiotherapy, and two more cycles of chemotherapy were administered after FSRT. Given this, there was a nine-week gap between the FSRT and the commencement of alectinib (at 600 mg twice daily). However, there are currently no clear guidelines regarding the acceptable interval between radiotherapy and second-generation TKIs.

**Figure 2:**
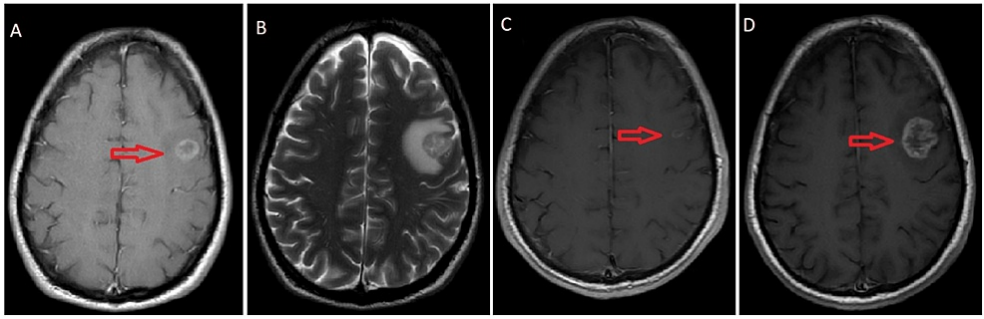
MRI brain images: A and B: pre-treatment brain MRI, T1- and T2-weighted contrast-enhanced; C: follow-up MRI of the brain four months post-FSRT; D: follow-up MRI of the brain one year post-FSRT shows interval progression in the size of the left frontal lesion compared to the post-FSRT response scan. FSRT: fractionated stereotactic radiotherapy

Follow-up MRI brain post-FSRT showed marked regression in the size of the left middle frontal gyrus enhancing lesion with regression of edema (Figure 2). Therefore, the patient was continued on TKI. A CT chest evaluation in February 2022 showed a further decrease in the size of the mass lesion in the left lower lobe.

In August 2022, the patient presented with seizures. The MRI brain showed interval progression in the size of the left frontal lesion compared to the post-FSRT response assessment scan and with disproportionate edema (Figure 2). Magnetic resonance spectroscopy (MRS) was inconclusive. As a result, the patient was referred for salvage brain RT. Assessment of the primary disease by CT scan revealed stable disease.

The patient was prescribed steroids and antiepileptics. The patient developed one episode of generalized tonic-clonic seizure (GTCS), and a repeat MRI of the brain showed a modest reduction in the edema while the enhancing lesions showed an interval increase in size.

**Figure 3:**
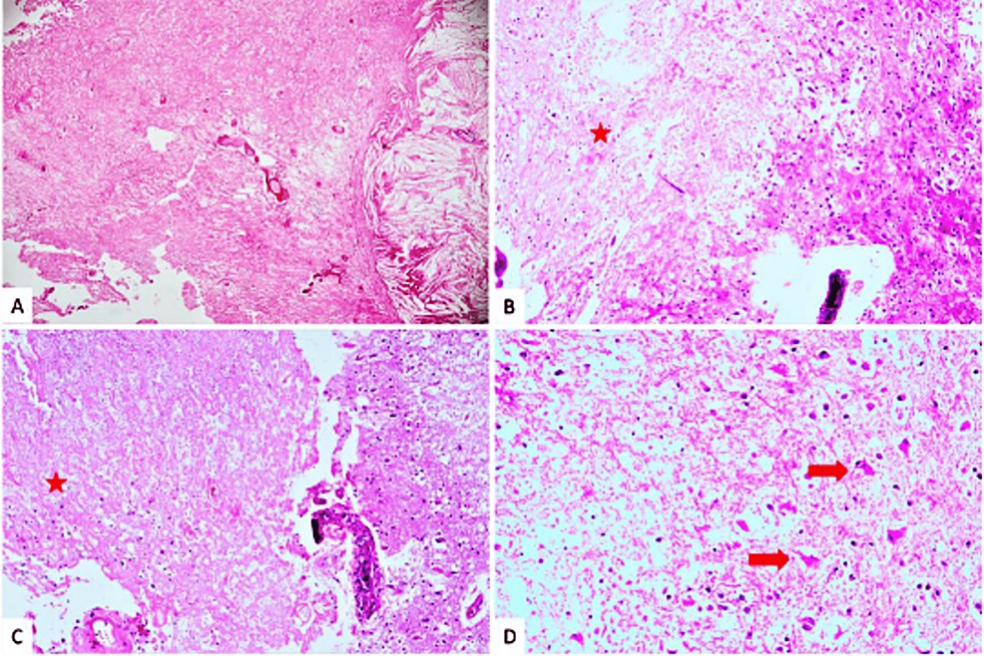
Postoperative histopathological examination slides gliotic brain parenchyma with treatment effects including necrosis. A: large area of necrosis with a focal collection of cholesterol clefts (H&E, magnification 4X); B: necrosis (red star) with adjacent gliotic brain parenchyma (H&E, magnification 10X); C: necrosis (red star) with gliotic brain parenchyma and blood vessel with perivascular lymphocytic cuffing (H&E, magnification 10X); D: necrosis and gliotic brain parenchyma with radiation-induced bizarre astrocytes (red arrows) (H&E, magnification 20X). H&E: hematoxylin and eosin

The index of suspicion for RN was high given that there were no new metastatic lesions in the brain for a relatively stable primary disease. Upon referral to a neurosurgeon, the patient underwent a decompression and excision biopsy in November 2022. Postoperative histopathological examination (HPE) revealed gliotic brain parenchyma with radiation-induced bizarre astrocytes, large areas of necrosis, and focal perivascular lymphocytic cuffing (Figure 3). In addition, leucocyte common antigen immunostains highlight the lymphocytes, and cytokeratin immunostains were negative and suggestive of RN.

## Discussion

The second-generation ALK inhibitor alectinib is not a substrate for p-glycoprotein and is, therefore, not eliminated from brain tissue through efflux. Moreover, a significant concentration of alectinib was detected in patients' cerebrospinal fluid (CSF) in a phase-1 clinical trial [13]. Increasingly, patients are offered upfront second-generation TKIs like alectinib owing to their robust CNS activity in asymptomatic brain metastases in ALK-positive NSCLC. However, CNS efficacy results from the phase III ALEX trial suggested that prior radiotherapy had a favorable intracranial overall response rate of over 75% in TKI-naive patients with measurable brain disease [14].

In the literature, FSRT with total doses of 24-35 Gy in three to seven fractions to patients with oligo-brain metastases resulted in an RN risk of approximately 2%-10% [15]. RN is a potentially debilitating complication of radiotherapy, especially radiosurgery, and can occur between three months and several years following treatment. Our patient had a solitary brain metastasis measuring approximately 2 cm in size. Considering the high risk of RN associated with the ALK-rearranged metastasis in our patient, FSRT was used to improve the therapeutic ratio. The BED for a single fraction of 18-20 Gy is approximately 180-220 Gy (for an α/β =2), while the BED for 35 Gy in 5 fractions is calculated to be 157 Gy (for an α/β=2). Moreover, based on consensus from the Netherlands for SRS dose prescriptions, a single dose of 24 Gy is prescribed for PTV sizes <1 cm3 and the dose level is stepwise decreased to 21, 18, and 15 Gy for PTV sizes between 1-10 cm3, between 10-20 cm3, and >20 cm3, respectively [16].

Further dose reduction may have reduced our patient's brain RN risk, although currently, the safe dose for ALK-rearranged brain metastases is unknown. Furthermore, the time gap between fractions for our patient was only 24 hours. Therefore, theoretically, FSRT schedules allowing a longer interval between consecutive doses would reduce the likelihood of RN compared to closely timed schedules. However, it is essential to note that the case presented in this report is a single instance. Thus, prospective studies must standardize the fractionation scheme, total radiation dose, and time interval between fractions to avoid brain RN, especially in ALK-rearranged brain metastases.

Our patient became symptomatic for brain RN 12 months post-FSRT while on alectinib. For metastases larger than 1 cm, a volumetric study comparing FSRT and SRS reported a 12-month risk of RN to be 3.9% versus 21.2%, while local control was 71% versus 47.7%, respectively [11]. Andruska et al. recently proposed residual V25Gy less than 16 cm3 and V30Gy less than 10 cm3 as dosimetric RN predictors [17]. Our patient's dose to the surrounding normal brain was within tolerance limits (Table 1).

**Table 1:** Technical characteristics of the plan and dose parameters PTV: planning target volume; Gy: Gray; cm^3^:cubic centimeter; VX Gy: volume receiving X Gy dose (brain-GTV); NA: not applicable; HI: D2%-D98%/DP; CI_Paddicks_:TV_PIV_^2^/(TV x VRI); GI: V50/PIV: D2%, D98% are the doses received by 2 %, 98% of the PTV and TV, VRI, TVPIV are the volumes of PTV, total volume covered by prescription isodose, target volume covered by the prescription isodose, V50-50% isodose volume.

Parameter	Target values	Achieved values
PTV Volume [cm^3^]	NA	7.78
Dose fractionation	NA	35 Gy in 5 fractions
Conformity index (CI)	0.9-1	0.95
Homogeneity index (HI)	0.0-0.1	0.024
Gradient index (GI)	<6	4.7
Brain-PTV [cm^3^]	NA	1419
V30 Gy [cm^3^]	< 10	8.84
V25 Gy [cm^3^]	< 16	15.5

The diagnosis of RN is based mainly on treatment history, symptoms, and imaging. However, imaging modalities alone are neither sensitive nor specific to identify RN, and despite utilizing multiparametric imaging in lower volume centers, it is still challenging to differentiate between RN and tumor progression [18].

In the literature, other case reports on RN in ALK-rearranged NSCLC with brain metastases are shown in Table 2. In 2015, Ou et al. reported two patients with RN presenting as pseudo-progression during alectinib therapy who had previously radiated brain metastases in ALK-positive NSCLC. These patients met the Response Evaluation Criteria in Solid Tumors (RECIST) criteria for progressive disease and had no extracranial progression; similar to our patient, both were initiated on Alectinib within four months of completing stereotactic radiosurgery [19]. In a subsequent study, the same authors reported a patient with alectinib-induced CNS RN in an ALK-positive NSCLC with a remote (seven-year) history of WBRT and SRS to right frontal lobe disease progression eight months post-WBRT [20].

**Table 2:** A literature review of case reports on radiation necrosis in ALK-rearranged brain metastases after radiation SRS: stereotactic radiosurgery; WBRT: whole brain radiotherapy; FSRT: fractionated stereotactic radiotherapy; ALK: anaplastic lymphoma kinase; TKI: tyrosine kinase inhibitors; NA: not available

Authors	Publication year	No. of patients	Radiation technique	ALK-TKI given	Time from RT to RN (months)
Ou et al. [19]	2015	2	SRS	Alectinib	NA
Ou et al. [20]	2016	1	WBRT+SRS	Alectinib	84
Song et al. [21]	2018	1	WBRT+SRS	Ceritinib	12
Zhu et al. [22]	2020	2	WBRT+SRS	Lorlatinib (multiple sequential TKI)	Case 1 - 7, Case 2 - 28
Present report	2023	1	FSRT	Alectinib	12

Song et al. described a patient with stage-IV ALK-positive NSCLC and three brain metastases who received WBRT 20 Gy in five fractions and commenced on ALK-TKI ceritinib in a clinical trial. Six months after WBRT, imaging studies demonstrated an increase in the size of the previously treated brain metastases with no new lesions. In addition, since the patient showed stable extracranial disease with good performance status, she was given a single fraction of SRS, 21 Gy, to the three brain lesions. Following the SRS patient's presentation with seizures, imaging, and clinical history reviewed, an RN was suggested. Their article emphasized that diagnosing RN is challenging, and differentiating it from tumor progression is a diagnostic dilemma [21]. Like our patient, diagnosis can significantly affect treatment decision-making, affecting the patient's overall outcomes.

Recently, Zhu et al. reported two patients who developed symptomatic brain RN while on treatment with lorlatinib for ALK-rearranged NSCLC, necessitating neurosurgical intervention [22]. Based on literature retrieval, this is the first published case report from Oman on FSRT for ALK-positive brain metastases. Currently, it is unclear what the risks of individual second-generation ALK-TKI are and how they may vary depending on the type of radiation delivered (total dose and fractionation schedule), the timing, and the sequencing of administration.

## Conclusions

The present case report indicates that, even with FSRT, further dose reduction is necessary to avoid brain RN. As the old adage goes, "the dose makes the poison," this would apply to SRT in ALK-rearranged cancer metastases of the CNS. Prospective trials would provide insight into what fractionation and radiation dose are safe in this group of patients receiving second-generation TKIs such as alectinib.
